# Deciphering molecular responses to radiofrequency microneedling in a 3D human skin model: new insights and modulation by aftercare treatment

**DOI:** 10.3389/fmed.2026.1799214

**Published:** 2026-04-22

**Authors:** Sebastian Huth, Yvonne Marquardt, Laura Huth, Sina Djahed, Jens Malte Baron

**Affiliations:** 1 Department of Dermatology and Allergology, Uniklinik RWTH Aachen, Aachen, Germany; 2 Djahed Aesthetics, Erkelenz, Germany; 3 Interdisciplinary Center for Laser Medicine, Uniklinik RWTH Aachen, Aachen, Germany

**Keywords:** aftercare, dexpanthenol, *in vitro* 3D skin model, microneedling, radiofrequency microneedling

## Abstract

**Background:**

Radiofrequency microneedling (RFMN) is an emerging therapeutic approach that enhances skin rejuvenation by delivering targeted thermal injury to the dermal and subdermal layers while sparing the epidermis. However, the molecular mechanisms underlying its biological effects especially in comparison to medical microneedling remain poorly understood. In this study, we aimed to investigate, for the first time, the molecular and histological responses to RFMN using a standardized three-dimensional (3D) human skin model. As a secondary objective, we assessed the modulatory effects of RFMN aftercare treatment with a dexpanthenol-containing ointment.

**Methods:**

We investigated the molecular and histological responses to RFMN utilizing a previously established standardized human 3D skin model composed of primary dermal fibroblasts and epidermal keratinocytes. Histological assessments and transcriptomic analyses employing next-generation sequencing were conducted 48 and 120 h after RFMN treatment. In addition, the effects of a topical dexpanthenol-containing ointment administered immediately following RFMN on wound healing and gene regulation were evaluated.

**Results:**

Histological analysis of RFMN-treated 3D skin models showed dermal coagulation zones that disappeared after post-treatment with a dexpanthenol-containing ointment. Transcriptomic profiling 48 h after RFMN showed an early pro-inflammatory response with upregulated chemokines and cytokines (e.g., CCL7, CXCL2, IL24) and downregulated matrix metalloproteinases and barrier genes, indicating controlled inflammation and skin renewal initiation. Aftercare with the dexpanthenol-containing ointment elicited distinct gene expression changes at 120 h post-RFMN, including increased expression of cytokines (IL37), matrix metalloproteinases (MMP3, MMP8), and epidermal regeneration markers (LCE1B, VEGFA), consistent with accelerated wound healing, re-epithelialization, and inflammation modulation.

**Conclusion:**

In conclusion, this study elucidates the molecular and histological effects of RFMN and highlights the benefits of dexpanthenol-containing topical post-treatment, which may further shorten the already brief downtime. These findings support the optimization of clinical protocols for safer and more effective skin rejuvenation.

## Introduction

Radiofrequency microneedling (RFMN) is a minimally invasive dermatological intervention suitable for all skin types, designed to address a broad range of indications in facial aesthetics, including skin rejuvenation, acne scarring, melasma, hair thinning, as well as dermatological conditions such as acne vulgaris and rosacea (1, 2). RFMN combines mechanical needle penetration of the skin with delivery of low-frequency electromagnetic energy into the lower dermis via insulated microneedles (3). The resulting localized thermal stimulation induces neocollagenesis and tissue remodeling while sparing the epidermis from significant injury due to lesser skin penetration in contrast to classical medical microneedling (2, 4). In addition to its thermal effects, the mechanical penetration of the epidermis and dermis by the microneedles enhances transdermal absorption of topically applied agents and promotes the release of growth factors (1, 2, 5). These mediators stimulate the migration and proliferation of keratinocytes and fibroblasts, thereby further supporting skin remodeling (6). Although the molecular effects of microneedling alone have been investigated in both animal tissue and human 3D skin models (5, 7, 8), the specific molecular mechanisms underlying the combined thermal and mechanical stimulation induced by RFMN are not fully understood. Recently, the U. S. Food and Drug Administration (FDA) has indicated potential risks associated with the use of RFMN devices, including burns and scarring, and has emphasized that these procedures are medical treatments requiring appropriate use (9). This highlights the importance of better understanding the precise mechanisms of action of these devices.

Post-procedure care plays a critical role in cosmetic dermatology, contributing to the prevention of adverse events and the promotion of optimal healing outcomes (10–12). However, it remains unclear whether the application of topical wound-healing agents following RFMN modulates or enhances its biological effects or reduces unwanted side effects of the RFMN treatment.

The aim of the present study was to investigate the molecular effects of RFMN using a standardized human *in vitro* full-thickness 3D skin model and to assess whether post-treatment with a dexpanthenol-containing ointment supports or modulates RFMN-induced responses.

## Materials and methods

### Isolation and cultivation of normal human epidermal keratinocytes (NHEK) and normal human dermal fibroblasts (NHDF)

Primary NHDF and NHEK cells were isolated and cultivated as described previously (13, 14). The collection and experimental use of human skin cells from donors was approved by the Ethics Committee of the Medical Faculty of RWTH Aachen University, Germany, in accordance with the principles of the Declaration of Helsinki. Written informed consent was obtained from all donors.

### 3D skin models

Full-thickness 3D skin models were generated as previously described (13). Briefly, the dermal compartment was constructed using a collagen-elastin scaffold (MatriDerm®; MedSkin Solutions Dr. Suwelack AG), which was cut into 19 mm circular punches and placed into six-well cell culture inserts (BD Falcon, Bedford, MA, USA). A total of 2 × 10^5 NHDF were seeded in a mixture of bovine collagen I solution (Vitrogen, Cohesion Technologies, Palo Alto, CA, USA) and 10x concentrated Hank’s balanced salt solution (Gibco/Invitrogen, Darmstadt, Germany) onto each scaffold and submerged in fibroblast growth medium. After 3 hours, 2 × 10^6 NHEK were seeded on top of the dermal equivalents. Two days later, the skin models were raised to the air-liquid interface (ALI). RFMN treatment was applied after the 3D skin models achieved complete epidermal differentiation.

### RFMN treatment of 3D skin models

**Figure 1:**
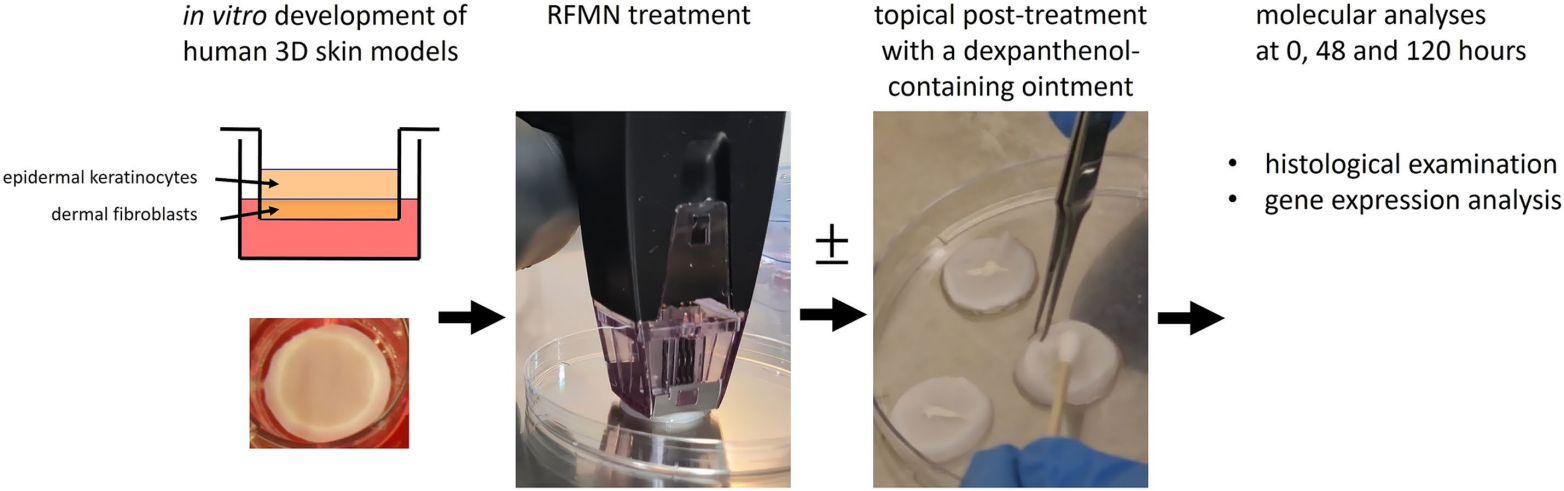
Schematic overview of the study design. Standardized *in vitro* 3D full-thickness skin models were generated using primary fibroblasts and keratinocytes. Subsequently, the models were treated with RFMN. In an additional experimental approach, models were topically post-treated with a dexpanthenol-containing ointment immediately following RFMN treatment. Untreated models served as controls. Histological assessments and transcriptomic analyses employing next-generation sequencing were conducted 48 and 120 h after RFMN treatment. All experiments were conducted twice in triplicate.

3D skin models were treated with RFMN using the Morpheus8 handpiece with 24-pin microneedle tips (InMode aesthetics, Düsseldorf, Germany). The penetration depth was 0.5 mm using energy level 8, according to the manufacturer’s and clinical treatment recommendations. Immediately after RFMN part of the models received aftercare treatment with a dexpanthenol-containing ointment (Bepanthen wound and care ointment, Bayer vital GmbH, Leverkusen Germany). After treatment, the models were cultivated in fresh culture medium and harvested on days 2 and 5 for histological analysis and detection of gene expression. Untreated models were maintained as negative controls. All experiments were performed in triplicate for every time point. A schematic overview of the study design is shown in Figure 1.

### Next-generation sequencing (NGS)

NGS analyses were performed as previously described (15). In brief, total RNA from 3D skin models was extracted using the NucleoSpin RNA Kit (Macherey-Nagel, Düren, Germany).

FASTQ files were generated using *bcl-convert* (Illumina, San Diego, USA), with demultiplexing performed based on a custom sample sheet and without lane splitting. Subsequent processing was carried out using the nf-core/rnaseq pipeline (v3.18.0), executed via Nextflow (v24.10.5). Reads were trimmed using Trim Galore (v0.6.10) and aligned to the human reference genome (hg38) with STAR (v2.7.11b). Gene-level quantification was performed using featureCounts, with grouping by *gene_type*. Differential expression analysis was conducted using DESeq2 (v1.42.1) within R (v4.3.3) using default parameters. The resulting differential gene expression data were further subjected to over-representation analysis (ORA) and gene set enrichment analysis (GSEA) using the clusterProfiler package (v4.10.1) in conjunction with the Gene Ontology (GO) database.

### Light microscopy

For light microscopy, 5 μm cryosections of 3D skin models were embedded in Tissue-Tek O. C. T.™ compound (Sakura Finetek), stained with hematoxylin and eosin (H&E), and examined using a photomicroscope (DMIL, Leitz, Wetzlar, Germany).

### Statistical analysis

Two independent experiments were performed, each in triplicate. For the analysis of the NGS data, genes were selected based on a log₂ fold change ≥ 1.5 and an adjusted *p* value < 0.05.

## Results

**Figure 2:**
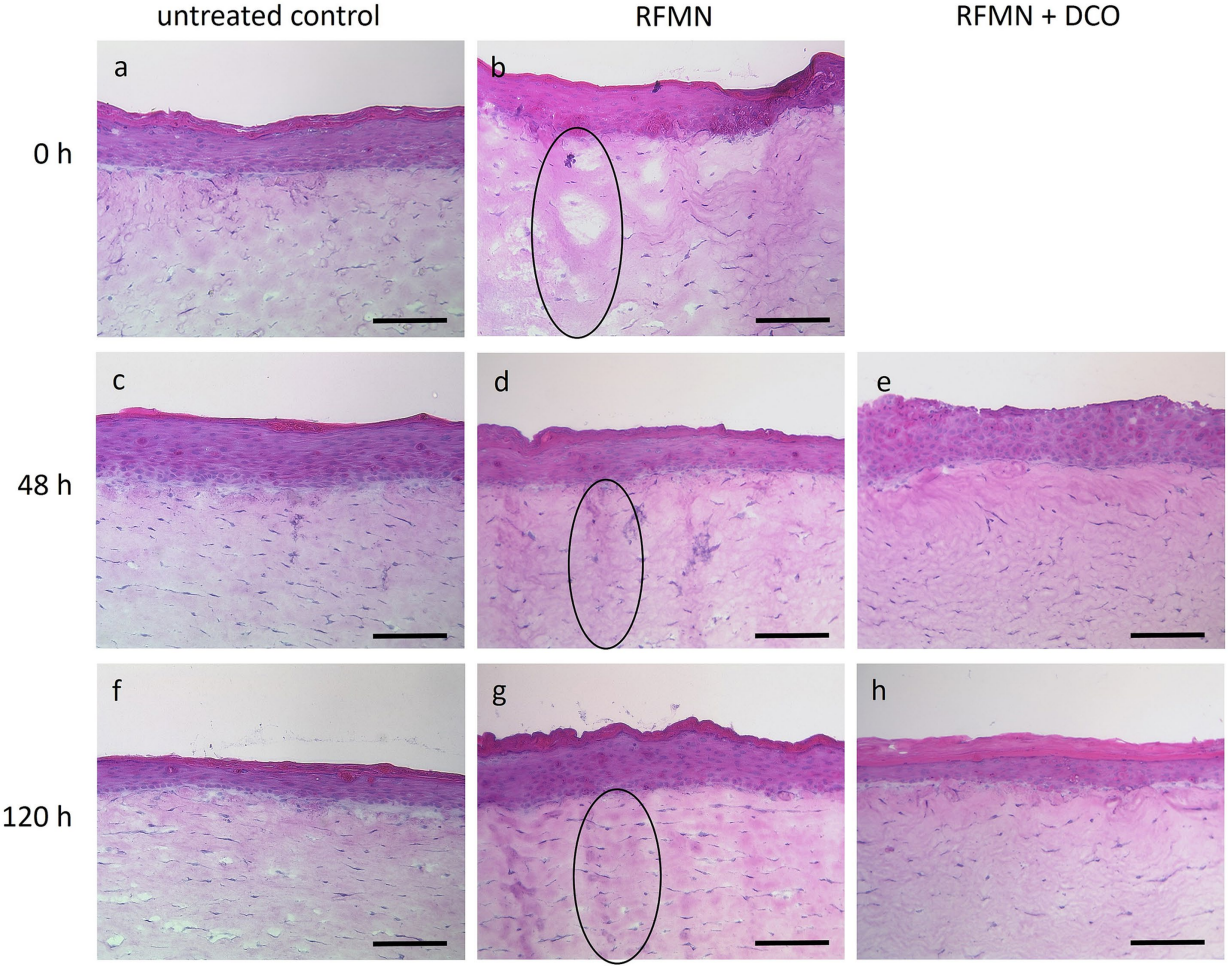
Hematoxylin and eosin stained cross-sections of **(a)** untreated 3D skin models, **(b)** 3D skin models immediately after RFMN, **(c)** untreated controls after 48 h, **(d)** 3D skin models 48 h after RFMN, **(e)** 3D skin models 48 h after RFMN following post-treatment with DCO, **(f)** untreated controls after 120 h, **(g)** 3D skin models 48 h after RFMN, and **(h)** D skin models 120 h after RFMN following post-treatment with DCO are shown. Representative images from two independent experiments, each performed in triplicate, are presented. Selected coagulation zones are exemplarily highlighted using ellipses. DCO = dexpanthenol-containing ointment. Magnification = 100×, scale bar = 200 μm.

Histological examination of cross-sections from 3D skin models treated with RFMN revealed dermal coagulation zones immediately following treatment (0 h; Figure 2b), which were absent in untreated controls (Figure 2a). These coagulation zones, indicative of thermal injury, remained clearly detectable at 48 and 120 h post RFMN treatment (Figures 2d,g), compared to untreated controls (Figures 2c,f). In contrast, 3D skin models treated with a dexpanthenol-containing ointment following RFMN exhibited less visible dermal coagulation zones at 48 and 120 h (Figures 2e,h). Notably, the epidermal layer of all models showed no significant visible damage at any time point following RFMN treatment.

**Figure 3:**
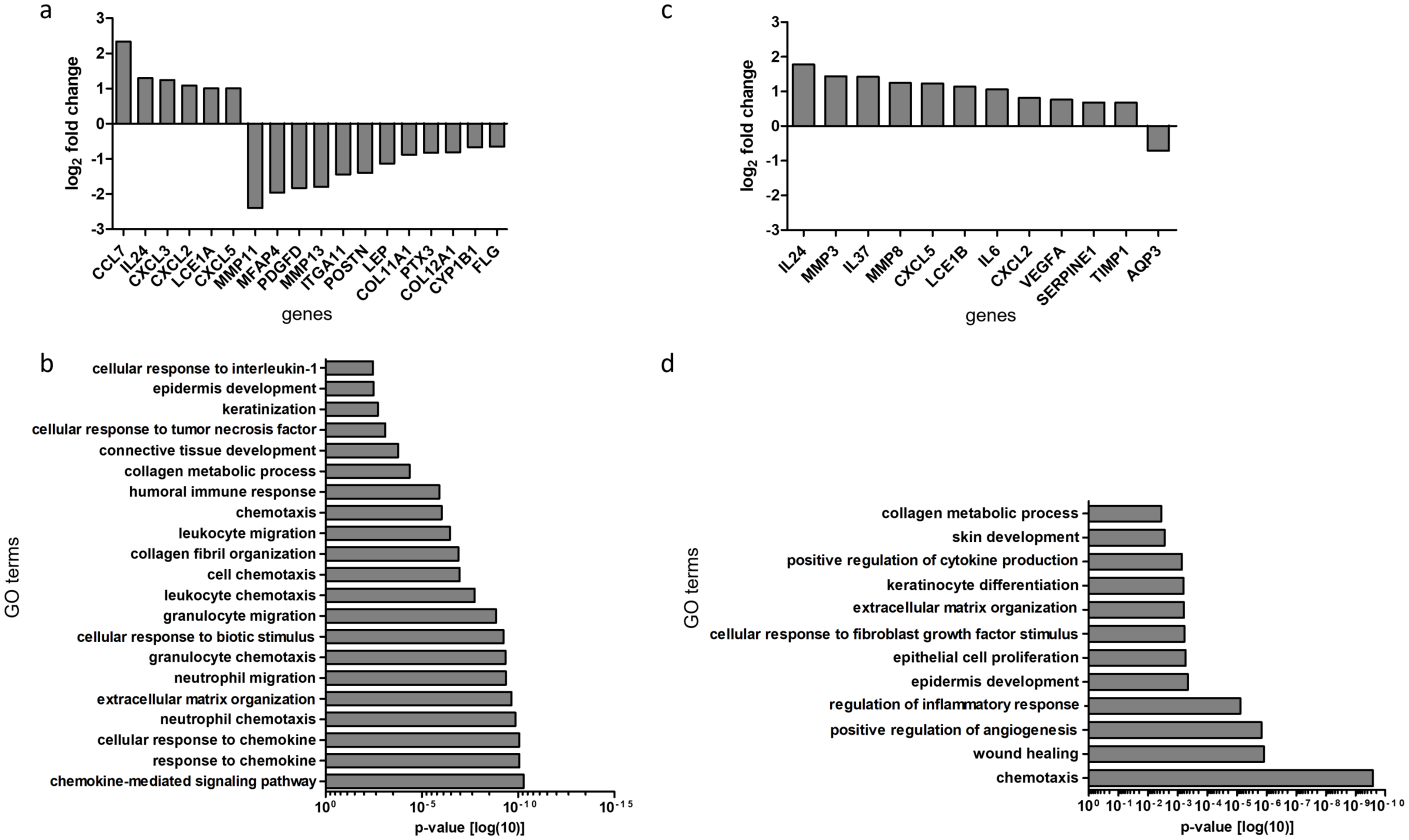
Gene expression profiling of RFMN-induced molecular effects. **(a)** Differential gene expression analysis of transcripts significantly regulated 48 h after RFMN compared to untreated controls (log₂ fold change > 1.5; adjusted *p* < 0.05). The bars represent the mean of a technical triplicate. **(b)** Gene ontology (GO) enrichment analysis of differentially expressed transcripts showing a significant overrepresentation of biological processes 48 h after RFMN compared to untreated controls. **(c)** Differential gene expression analysis of transcripts significantly regulated 120 h after RFMN with subsequent post-treatment using a dexpanthenol-containing ointment, compared to RFMN-treated 3D skin models without post-treatment (log₂ fold change > 1.5; adjusted *p* < 0.05). **(d)** GO enrichment analysis of differentially expressed transcripts revealed a significant overrepresentation of biological processes 120 h after RFMN followed by post-treatment with a dexpanthenol-containing ointment, compared to RFMN-treated 3D skin models without post-treatment.

Next, we aimed to investigate the molecular effects of RFMN treatment on our 3D skin models using NGS. At 48 h post-RFMN treatment, differential gene expression analysis revealed a distinct set of transcripts significantly regulated compared to untreated controls (Figure 3a). Several chemokines and cytokines, including *CCL7*, *CXCL2*, *CXCL3*, *CXCL5*, and *IL24*, exhibited significant upregulation (log2 fold-change > 1.5; adjusted *p* < 0.05). Conversely, downregulation was observed for matrix metalloproteinases (*MMP11*, *MMP13*), skin barrier markers (*FLG*), genes associated with wound closure (*POSTN*, *PTX3*, *ITGA11*, *LEP*, *PDGFD*), and collagens (*COL11A1*, *COL12A1*).

To date, data on the biological processes and pathways affected by RFMN remain limited. On day 2 post-treatment, Gene Ontology (GO) enrichment analysis of differentially expressed transcripts demonstrated a significant overrepresentation of biological processes related to tissue remodeling and skin regeneration, including “collagen metabolic process”, “collagen fibril organization”, “extracellular matrix organization”, “keratinization”, and “epidermis development” (Figure 3b). Additionally, inflammatory processes such as “chemotaxis”, “cellular response to chemokine”, “neutrophil chemotaxis”, and “chemokine-mediated signaling pathway” were significantly enriched. A comparative analysis between RFMN-treated models with and without subsequent post-treatment using a dexpanthenol-containing ointment revealed no statistically significant differences in gene expression profiles at day 2 post-treatment. By day 5 post-treatment, comparison of RFMN followed by post-treatment with a dexpanthenol-containing ointment versus RFMN alone revealed a significant upregulation of cytokines and chemokines (*IL24*, *IL37*, *CXCL5*, *IL6*, *CXCL2*), matrix metalloproteinases (*MMP3*, *MMP8*), and genes involved in epidermal differentiation and wound healing (*LCE1B*, *VEGFA*, *SERPINE1*) (Figure 3c). Correspondingly, GO enrichment analysis indicated significant involvement of biological processes related to “wound healing,” “epidermis development,” “keratinocyte differentiation,” “extracellular matrix organization,” and “regulation of inflammatory responses” (Figure 3d). A comparison between RFMN-treated samples and untreated controls showed no significant differences in gene expression at this time point.

## Discussion

The combination of microneedling with radiofrequency technology represents a novel therapeutic modality that enhances skin rejuvenation by inducing targeted heating within the dermal, subdermal, and adipose compartments, while concurrently limiting epidermal trauma (16). Previous studies investigating the effects of RFMN have often used *in vivo* human or animal skin (8, 17, 18), which is ethically controversial and offers only limited applicability, as the biological properties of laboratory animal skin differ from those of human skin. To the best of our knowledge, this study presents the first analysis of both histological and molecular responses to RFMN using *in vitro* 3D skin models and explores the potential benefits of aftercare with a wound-healing-promoting topical dexpanthenol-containing ointment. In previous studies, we have already investigated the molecular effects of conventional medical microneedling in such *in vitro* 3D skin models (5, 7), thereby enabling now a direct comparison between classical microneedling and RFMN. The investigation time points of 48 and 120 h selected in our study were chosen because they best capture the phases of wound healing and remodeling in our in vitro models, while also allowing for direct comparability with our previous studies on conventional medical microneedling (5, 7).

Histologically, the presence of dermal coagulation zones in the 3D skin models immediately following RFMN treatment confirms that the procedure induced localized thermal injury within the dermal equivalents, consistent with previously reported structural changes observed in RFMN-treated tissues (17, 18). The persistence of these zones up to 120 h post-treatment indicates sustained tissue remodeling and healing activity in the dermal compartment. Importantly, the absence of visible damage to the epidermal layer highlights that RFMN selectively targets the dermis, thereby minimizing superficial epidermal disruption, as previously described in the literature (1, 4, 19). This dermis-specific targeting is clinically relevant, as it is associated with a shorter recovery period and a reduced risk of epidermal barrier impairment and related complications, such as infection or delayed wound healing. It also explains why RFMN has less effect on skin pigmentation and can be applied clinically in different skin types (I-VI) (3).

The significance of appropriate post-procedural care in cosmetic dermatology for minimizing adverse events and enhancing clinical outcomes is well established (10, 11). In our previous studies, we demonstrated that topical aftercare with a dexpanthenol-containing ointment facilitates accelerated cutaneous wound healing following medical microneedling or ablative laser irradiation of 3D skin models and enhances the post-procedural care regimen, thereby potentially minimizing patient downtime (7, 13–15, 20). In our present study, histological analysis based on a qualitative comparison of the histological images showed fewer coagulation zones in 3D skin models after RFMN when post-treated with a dexpanthenol-containing ointment. This finding suggests an accelerated recovery of dermal thermal injuries. Collectively, these results highlight the potential of a wound-healing-promoting topical aftercare treatment to enhance tissue recovery after RFMN and to further minimize the short downtime typically observed with this procedure.

While RFMN is recognized for its ability to promote dermal remodeling - primarily through the stimulation of collagen, elastin, and hyaluronic acid synthesis via radiofrequency energy delivery (21), the underlying molecular mechanisms remain poorly understood. In our study, transcriptomic analysis using NGS provides insights into the dynamic gene expression changes induced by RFMN. In particular, a marked upregulation of proinflammatory chemokines and cytokines was observed 2 days after RFMN treatment, presumably due to the stress-induced response of the keratinocytes. These molecules are essential for recruiting immune cells, such as neutrophils, to the site of tissue injury, thereby initiating the inflammatory phase of wound healing - an indispensable first step in triggering subsequent repair and regeneration (11). The upregulated expression of cytokines and chemokines may be explained by activation of NF-κB, a key regulator of the inflammatory response (22). The concomitant downregulation of matrix metalloproteinases and genes related to skin barrier integrity and wound closure after RFMN likely corresponds to the early inflammatory phase of wound healing, preceding the transition into the proliferative and remodeling phases (23). Gene Ontology enrichment analysis complements these findings by demonstrating overrepresentation of biological processes central to tissue repair, including “collagen metabolism” and “fibril organization,” “extracellular matrix organization,” “keratinization,” and “epidermis development.” These terms are likely overrepresented due to the significant downregulation of the corresponding involved genes and reflect the initiation of the wound healing cascade. The enrichment of “chemotaxis” and “chemokine-mediated signaling pathways” reinforces the critical role of immune cell recruitment and inflammatory signaling in the early response to RFMN-induced dermal injury. Overall, these data are consistent with our previously conducted *in vitro* studies investigating the effects of medical microneedling on 3D skin models (5, 7). Although medical microneedling induced more pronounced epidermal injury in 3D skin models compared to RFMN, both treatments elicit similar molecular responses, particularly the activation of early pro-inflammatory and chemokine-dependent signaling cascades. Hence, RFMN triggers comparable molecular mechanisms to medical microneedling, while causing considerably less damage to the upper epidermal layers.

Two days following RFMN treatment, 3D skin models receiving aftercare with the dexpanthenol-containing ointment did not exhibit differential gene expression patterns compared to models treated with RFMN alone. This suggests that, despite clear histological differences, the effects of dexpanthenol may not yet significantly modulate the molecular responses induced by RFMN at this early time point. This changed 5 days after RFMN treatment, when 3D skin models receiving post-treatment with a dexpanthenol-containing ointment exhibited distinct transcriptional changes compared to those treated with RFMN alone. The upregulation of both pro-inflammatory cytokines and chemokines, such as IL24, IL6, CXCL5, and CXCL2, as well as anti-inflammatory cytokines including IL37, indicates a sustained but regulated inflammatory environment. This may favor the resolution of inflammation rather than its exacerbation. The observed induction of matrix metalloproteinases at this later stage of wound healing on day 5 after RFMN treatment may reflect active extracellular matrix remodeling, which is critical for wound closure and tissue regeneration (24). Furthermore, genes associated with epidermal differentiation and wound healing, such as LCE1B, VEGFA, and SERPINE1, were significantly upregulated. This observation aligns with previous studies investigating the effects of dexpanthenol-containing ointment as an aftercare treatment following medical microneedling and ablative laser treatments, thereby suggesting an enhancement of wound healing processes in response to topical dexpanthenol application (7, 13–15, 20). The observed gene expression changes are corroborated by GO enrichment analyses, which reveal significant engagement of pathways related to “wound healing,” “epidermis development,” “keratinocyte differentiation,” “extracellular matrix organization,” and “regulation of inflammatory responses.” These data suggest that the topically applied dexpanthenol-containing ointment not only promotes skin recovery but also modulates the inflammatory environment to support balanced tissue regeneration. This may potentially accelerate functional recovery. Collectively, our findings provide mechanistic insights into how RFMN initiates a controlled injury response that activates tissue remodeling and repair pathways, and how aftercare with a dexpanthenol-containing ointment may optimize these processes. Interestingly, the molecular effects on gene expression did not directly mirror the visible histological changes induced by RFMN treatment and subsequent post-treatment with the dexpanthenol-containing ointment. While RFMN treatment in our 3D skin models alone primarily elicited molecular effects at an early time point in the wound healing process on day 2, aftercare with the dexpanthenol-containing ointment induced molecular changes at a later stage, specifically on day 5. Generally, the wound healing process is described as comprising three overlapping phases – inflammation, proliferation and remodeling (11). The inflammatory phase begins immediately after injury and typically lasts 48–96 h (25). Thus, at the molecular level, RFMN seems to exert its effects primarily by acting on the inflammatory phase, while post-treatment with the dexpanthenol-containing ointment can further modulate this inflammatory phase and particularly influence the subsequent proliferative and remodeling phases.

Limitations of this study include the use of *in vitro* 3D skin models, which, while physiologically relevant, cannot fully recapitulate the complexity of *in vivo* skin responses, including systemic immune interactions and vascular dynamics. Nevertheless, such in vitro models provide an important contribution, particularly in accordance with the 3R principles (26), and enable molecular investigations over time courses that would not be feasible in human in vivo studies due to ethical considerations. Furthermore, these model systems, being derived from human skin cells, more accurately replicate the physiological characteristics of native human skin and thus offer a distinct advantage over studies utilizing animal skin.

In conclusion, this study advances our understanding of the molecular and histological effects of RFMN on skin tissue and underscores the beneficial impact of topical aftercare with a dexpanthenol-containing ointment, which may further reduce the already short downtime associated with RFMN. These findings may contribute to the development of improved clinical protocols for skin rejuvenation therapies, thereby optimizing both efficacy and safety. In comparison to previous studies analyzing molecular effects of medical microneedling in this 3D skin model, RFMN stimulated similar proinflammatory and chemotactic pathways leading to subsequent tissue remodeling without significant alteration of the upper epidermis. This might explain the benefits of using RFMN on darker skin types or sensitive skin areas such as the neck and décolleté.

## Data Availability

The datasets discussed in this study are available in Gene Expression Omnibus (GEO) database (https://www.ncbi.nlm.nih.gov/geo/) with accession number GSE327760.
